# Loss of *Pla2r1* decreases cellular senescence and age‐related alterations caused by aging and Western diets

**DOI:** 10.1111/acel.13971

**Published:** 2023-09-04

**Authors:** Amélie Massemin, Delphine Goehrig, Jean‐Michel Flaman, Sara Jaber, Audrey Griveau, Sophia Djebali, Elisabeth Marcos, Léa Payen, Jacqueline Marvel, Romain Parent, Serge Adnot, Philippe Bertolino, Jennifer Rieusset, Antonin Tortereau, David Vindrieux, David Bernard

**Affiliations:** ^1^ Centre de Recherche en Cancérologie de Lyon, Inserm U1052, CNRS UMR 5286, Centre Léon Bérard Université de Lyon Lyon France; ^2^ Equipe Labellisée la Ligue Contre le Cancer Lyon France; ^3^ Centre International de Recherche en Infectiologie, Inserm U1111, CNRS UMR5308, École Normale Supérieure de Lyon Université de Lyon, Université Claude Bernard Lyon 1 Lyon France; ^4^ INSERM U955, Département de Physiologie ‐ Explorations fonctionnelles, Hôpital Henri Mondor AP‐HP, FHU SENEC Créteil France; ^5^ Laboratoire de Biochimie et Biologie Moléculaire, Centre Hospitalier Lyon Sud Hospices Civils de Lyon Pierre Bénite France; ^6^ CarMeN Laboratory, UMR INSERM U1060/INRA U1397 Lyon 1 University Pierre bénite France; ^7^ VetAgro Sup, Interactions Cellules Environnement (ICE) Université de Lyon Marcy l'Etoile France

**Keywords:** aging, cellular senescence, liver, Western diet

## Abstract

Cellular senescence is induced by many stresses including telomere shortening, DNA damage, oxidative, or metabolic stresses. Senescent cells are stably cell cycle arrested and they secrete many factors including cytokines and chemokines. Accumulation of senescent cells promotes many age‐related alterations and diseases. In this study, we investigated the role of the pro‐senescent phospholipase A2 receptor 1 (PLA2R1) in regulating some age‐related alterations in old mice and in mice subjected to a Western diet, whereas aged wild‐type mice displayed a decreased ability to regulate their glycemia during glucose and insulin tolerance tests, aged *Pla2r1* knockout (KO) mice efficiently regulated their glycemia and displayed fewer signs of aging. Loss of *Pla2r1* was also found protective against the deleterious effects of a Western diet. Moreover, these *Pla2r1* KO mice were partially protected from diet‐induced senescent cell accumulation, steatosis, and fibrosis. Together these results support that *Pla2r1* drives several age‐related alterations, especially in the liver, arising during aging or through a Western diet.

## Graphical abstract

Loss of PLA2R1 improves the response of mice to an obesogenic diet or to chronological aging, probably by decreasing the accumulation of senescent cells.

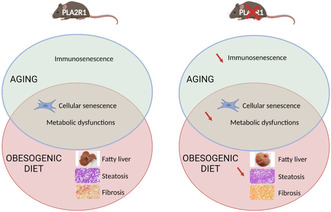

**Abbreviations**
ALP: alkaline phosphatase
CD: chow diet
COPD: chronic obstructive pulmonary diseases
DEGs: differentially expressed genes
FA: fatty acids
H&E: hematoxylin and eosin
HSCs: hepatic stellate cells
HSC: hematopoietic stem cells
IHC: immunohistochemistry
IPGTT: intraperitoneal glucose tolerance test
KO: knock‐out
NAFLD: nonalcoholic fatty liver disease
NASH: nonalcoholic steatohepatitis
PLA2R1: phospholipase A2 receptor 1
ROS: reactive oxygen species
SASP: senescence‐associated secretory phenotype
scRNA‐seq: single‐cell RNA‐sequencing
SPF: specific‐pathogen‐free
TG: triglycerides
WD: western diet
WT: wild‐type

## 1 INTRODUCTION

Cellular senescence is a form of stable cell proliferation arrest associated with the secretion of many soluble factors such as cytokines, chemokines, and proteases, known as a senescence‐associated secretory phenotype (SASP; Coppé et al., 2010). Cellular senescence was discovered for the first time in 1961 by Hayflick and Moorhead as replicative exhaustion due to telomere attrition (Hayflick & Moorhead, 1961; Victorelli & Passos, 2017). Other external and internal stimuli such as reactive oxygen species (ROS), oncogenes, chemotherapy, DNA damage, or mitochondrial dysfunction can also lead to premature senescence (Hernandez‐Segura et al., 2018). Senescent cell accumulation promotes age‐related diseases and aging. Aging and a poor lifestyle (cigarette smoke, obesity, etc.) are known to promote cell entry into senescence and senescent cell accumulation and to decrease senescent cell elimination (Baker et al., 2011; Campisi, 2012; Di Micco et al., 2021; Kowald et al., 2020). Elimination of senescent cells or controlling their SASP has become a major challenge in regulating many age‐related chronic diseases, including cardiovascular diseases, cancer, dementia, and metabolic disorders, to improve healthy aging (He & Sharpless, 2017). Nevertheless, cellular senescence also exerts beneficial effects such as blocking tumorigenesis or promoting wound healing (Demaria et al., 2014; He & Sharpless, 2017).

The incidence of metabolic disorders has increased over the last decades owing to a change in diet, higher in sugar and fat, and to a more sedentary lifestyle. These disorders include dysregulations in glucose metabolism and diabetes, hypertriglyceridemia found in obesity, and nonalcoholic fatty liver disease (NAFLD; Papatheodoridi et al., 2020; Schafer et al., 2017). NAFLD is one of the most common liver diseases affecting 20%–25% of the population worldwide and is the top‐ranking cause of liver disease in Western countries. NAFLD triggers a wide range of disorders from simple steatosis to steatohepatitis, related fibrosis, and hepatocellular carcinoma (Anstee et al., 2019; Bessone et al., 2019; Younossi et al., 2018). Increasing evidence supports a key role for senescent cells in promoting NAFLD, as their elimination or modulation can improve several hallmarks of nonalcoholic steatohepatitis (NASH; Papatheodoridi et al., 2020).

Several factors have been shown to regulate cellular senescence, most notably effectors directly blocking cell cycles, such as the p53 protein, or promoting the SASP, such as the NF‐κB transcription factor and the mTOR pathway. However, little is known about pro‐senescent signals at the membrane of cells that could constitute new markers to target and eliminate senescent cells or to alter their activity (Rossi & Abdelmohsen, 2021). The phospholipase A2 receptor 1 (PLA2R1), belonging to the C‐type lectin superfamily and the mannose receptor family (East & Isacke, 2002), has been identified as a pro‐senescent protein (Augert et al., 2009; Beaulieu et al., 2021; Griveau et al., 2018; Vindrieux et al., 2013). It also promotes premature age‐related phenotypes during progeria and accelerates emphysema and fibrosis in the lung in the context of chronic obstructive pulmonary diseases (COPD), probably through the induction of senescence (Beaulieu et al., 2021; Griveau et al., 2018).

In this study, we sought to determine whether the senescence regulator PLA2R1 may impact some age‐related phenotypes in aging mice. Our results support that *Pla2r1* knockout (KO) mice display fewer signs of aging and enhanced regulation of glucose homeostasis. These observations were confirmed in *Pla2r1* KO mice subjected to a Western diet (WD), as they displayed a better ability to regulate glucose homeostasis, as well as less liver steatosis, fibrosis, and marks of cellular senescence compared with wild‐type (WT) littermates.

## 2 RESULTS

### 2.1 *Pla2r1* knockout alleviates age‐related alterations and cellular senescence caused by aging and Western diets

*Pla2r1* is a well‐known pro‐senescent gene (Augert et al., 2009; Beaulieu et al., 2021; Griveau et al., 2018; Vindrieux et al., 2013) that fosters some age‐related phenotypes in COPD and progeria (Beaulieu et al., 2021; Griveau et al., 2018). Nevertheless, its role in physiological aging or in other age‐related alterations is unknown. Old *Pla2r1‐*deficient mice (21 months) and their WT controls were analyzed for several features of aging, such as immunosenescence, defined as aging of the immune system, systemic glucose intolerance, and insulin resistance (Chang & Halter, 2011), telomere shortening (Bernadotte et al., 2016) and liver alterations (Kim et al., 2015).

Immunosenescence is characterized by several parameters including an increase in cellular senescent immune cells, immune cell exhaustion, a decrease in the ratio of naive/memory T lymphocytes, a decrease in immune cell functionality and an increase in short‐term (ST)/ long‐term (LT) hematopoietic stem cells (HSC) ratio (Aiello et al., 2019; Lian et al., 2020; Rodriguez et al., 2021; Xu et al., 2020). Compared with old WT mice, we observed in old *Pla2r1* KO mice (i) a decrease in CD4+ T senescent cells (KLRG1+), (ii) a decrease in the exhaustion of CD8+ T cells (PD1+), (iii) a slight increase in naive/memory CD4+ T cell ratio, (iv) better functioning CD8+ T cells, as more produced IFNγ in response to stimulation by either CD3/CD28 or by PMA/ionomycin, and (v) a relative decrease in ST/LT HSC ratio (Table 1). Interestingly, these differences were not observed in young *Pla2r1* KO mice (Table 1), suggesting that loss of *Pla2r1* decreases marks of immunosenescence during mice aging.

**TABLE 1:** Old *Pla2r1* knock‐out mice display less signs of immunosenescence.

	Young			Old		
	WT	KO	*t* test	WT	KO	*t* test
CD45+ Leucocytes^a^						
Spleen	9,37E+7 ± 1,82E+7	8598E+7 ± 9,20E+6	0.675	8,18E+7 ± 8,18E+6	9,20E+7 ± 2,45E+6	0.258
Lymph node	2,11 E+6 ± 5,75 E+5	2,68E+6 ± 8,38E+5	0.650	5,44E+5 ± 1,35E+5	5,22E+5 ± 1,40E+5	0.887
CD3+ T lymphocytes^b^						
Spleen	25,50 ± 0,87	27,61 ± 1,56	0.361	15,80 ± 1,11	15,71 ± 0,85	0.951
Lymph node	49,55 ± 4,55	54,43 ± 3,18	0.392	43,82 ± 2,14	48,13 ± 3,86	0.351
CD4+ T lymphocytes^b^						
Spleen	13,38 ± 0,67	14,31 ± 1,01	0.534	9,37 ± 0,50	9,10 ± 0,51	0.714
Lymph node	22,78 ± 2,70	25,97 ± 1,79	0.332	18,83 ± 1,30	17,40 ± 1,56	0.496
CD4+ KLRG1+ T lymphocytes^c^						
Spleen	1,12 ± 0,06	1,01 ± 0,15	0.609	**1,77** **±** **0,25**	**1,07** **±** **0,07**	**0.037**
Lymph node	1,33 ± 0,11	1,39 ± 0,16	0.801	**2,40 ± 0,53**	**1,72 ± 0,15**	**0.267**
CD4+ PD1+ T lymphocytes^c^						
Spleen	4,14 ± 0,80	3,28 ± 0,46	0.338	39,08 ± 4,01	33,32 ± 2,67	0.259
Lymph node	1,97 ± 0,23	1,93 ± 0,21	0.842	17,47 ± 3,44	14,30 ± 1,33	0.411
Ratio CD4+ naive/memory T lymphocytes						
Spleen	2,61 ± 0,28	2,80 ± 0,27	0.663	**0,45 ± 0,11**	**0,59 ± 0,09**	**0.360**
Lymph node	4,72 ± 0,19	5,12 ± 0,43	0.519	**0,55 ± 0,12**	**0,75 ± 0,07**	**0.184**
CD8+ T lymphocytes^b^						
Spleen	9,75 ± 0,73	10,56 ± 0,74	0.494	6,41 ± 0,74	6,96 ± 0,47	0.545
Lymph node	24,86 ± 1,68	25,01 ± 1,24	0.946	24,98 ± 1,81	30,73 ± 2,70	0.107
CD8+ KLRG1+ T lymphocytes^d^						
Spleen	0,62 ± 0,13	0,54 ± 0,12	0.684	2,38 ± 0,40	2,44 ± 0,67	0.934
Lymph node	0,12 ± 0,03	0,15 ± 0,03	0.456	0,62 ± 0,15	0,49 ± 0,11	0.472
CD8+ PD1+ T lymphocytes^d^						
Spleen	1,12 ± 0,13	0,80 ± 0,11	0.109	**6,02** **±** **0,84**	**3,05** **±** **0,50**	**0.018**
Lymph node	0,30 ± 0,03	0,37 ± 0,04	0.260	**4,15 ± 1,55**	**1,98 ± 0,33**	**0.226**
Ratio CD8+ naive/memory T lymphocytes						
Spleen	1,71 ± 0,10	1,81 ± 0,12	0.586	0,48 ± 0,09	0,45 ± 0,05	0.832
Lymph node	3,07 ± 0,17	3,62 ± 0,23	0.126	0,65 ± 0,09	0,68 ± 0,09	0.821
B220+ CD19+ lymphocytes^b^						
Bone marrow	46,6 ± 3,92	43,57 ± 2,99	0.555	14,77 ± 1,44	16,80 ± 1,61	0.368
Ratio ST/LT HSC						
Bone marrow	‐	‐	‐	0,43 ± 0,10	0,28 ± 0,09	0.281
CD8 + IFNg + splenocytes after stimulation by^d^						
CD3/CD28	2,21 ± 0,45	2,84 ± 0,56	0.408	**6,86 ± 0,85**	**8,82 ± 0,19**	**0.069**
PMA/ionomycin	16,25 ± 1,45	15,08 ± 1,02	0.519	**27,88 ± 1,34**	**33,03 ± 2,92**	**0.140**

*Note*: Immunophenotyping of spleen, lymph node, and bone marrow of 3‐month‐old (young) wild type (WT; *n* = 7) and *Pla2r1* KO (*n* = 4) mice and of 21‐month‐old (old) WT (*n* = 6) and *Pla2r1* KO (*n* = 6) mice. The ratio ST/LT HSC corresponds to short‐term/long‐term ratio of hematopoietic stem cells. Mean ± SEM. Unpaired *t* test between WT and *Pla2r1* KO young mice and WT and *Pla2r1* KO old mice. The values in bold indicate a trend of difference and the values in bold and underlined indicate a significant difference.

^a^ The values represent the cell number of leucocytes.

^b^ The frequency of subpopulations to leucocytes.

^c^ The frequency of subpopulations to CD4+ T lymphocytes.

^d^ The frequency of subpopulations to CD8+ T lymphocytes.

**FIGURE 1:**
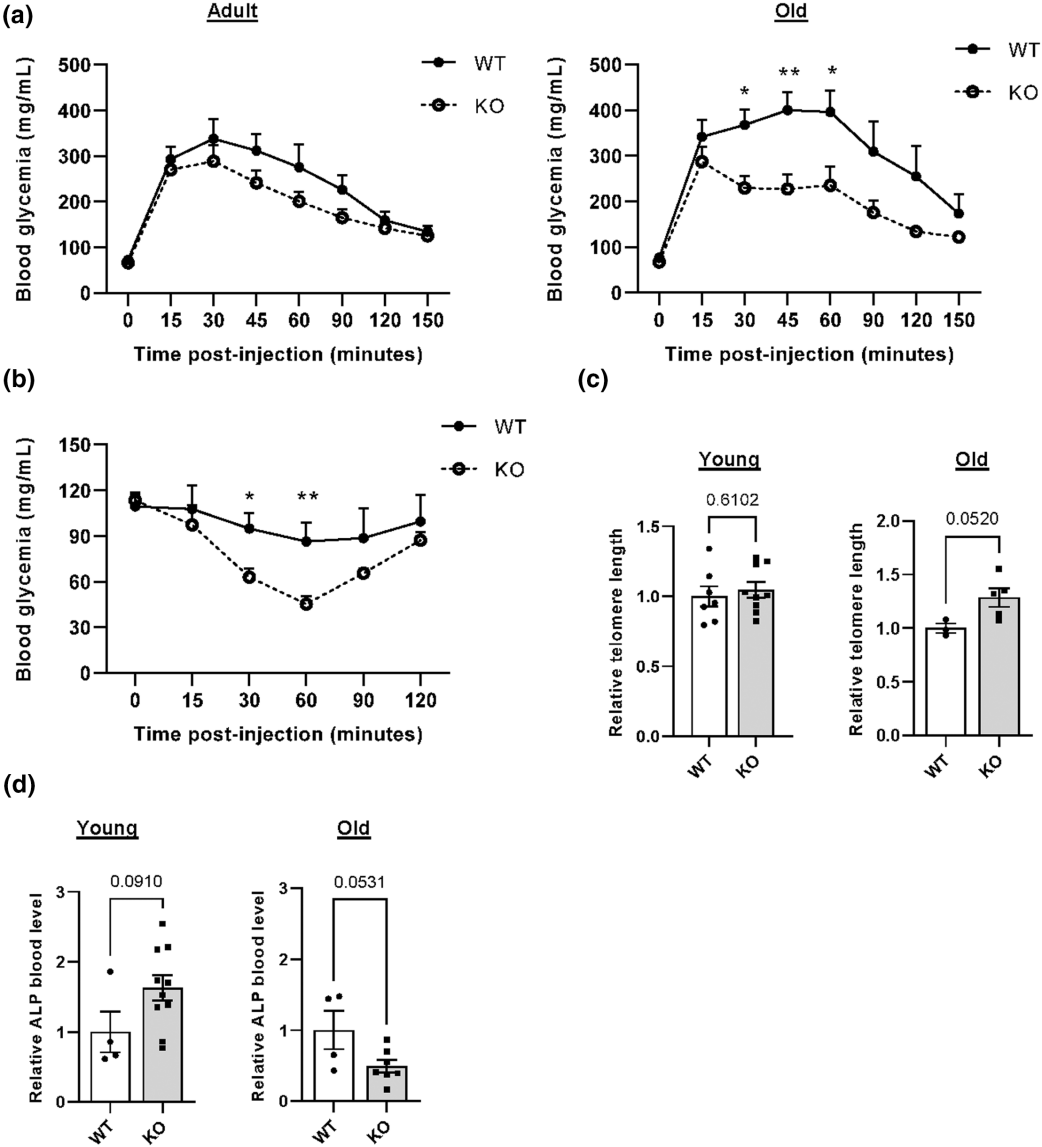
Old *Pla2r1* knockout (KO) mice display fewer age‐related marks. (a) Intraperitoneal glucose tolerance test and (b) intraperitoneal insulin tolerance test of 14‐month‐old (adult) WT (*n* = 7) and *Pla2r1* KO (*n* = 8; for a), and 20‐month‐old (old) WT (*n* = 7) and *Pla2r1* KO (*n* = 8; for a and b) female mice. Mean ± SEM. Multiple Mann–Whitney tests. *p < 0.05, **p < 0.01. (c) Relative telomere length of liver from 3‐month‐old (young) WT (*n* = 7) and *Pla2r1* KO (*n* = 9) and from 21‐month‐old (old) WT (*n* = 3) and KO *Pla2r1* (*n* = 5) female mice. Mean ± SEM. Unpaired two‐tailed Student *t* test. (d) Quantification of relative blood ALP level in WT (*n* = 4) and *Pla2r1* KO (*n* = 10) 3‐month‐old (young) mice, and WT (*n* = 4) and *Pla2r1* KO (*n* = 7) 21‐month‐old (old) mice. Mean ± SEM. Unpaired two‐tailed Student *t* test.

During aging, we also observed that old *Pla2r1* KO mice were leaner than WT mice, suggesting a healthier aging (Figure S1A). To study the functional impact of PLA2R1 on metabolism and glucose homeostasis during aging, intraperitoneal glucose tolerance tests (IPGTT) were performed on 20‐month‐old mice. Old *Pla2r1* KO mice displayed a better ability to normalize their glycemia than WT mice during IPGTT (Figure 1a, right panel), whereas no differences were observed in adult mice (Figure 1a left panel). Interestingly, while insulin production was similar between the two genotypes during IPGTT (Figure S1B), old *Pla2r1* KO mice more swiftly downregulated their glycemia following insulin injection during intraperitoneal insulin tolerance tests compared to old WT mice (Figure 1b). Therefore, old *Pla2r1* KO mice displayed a higher insulin sensitivity, leading to better control of glucose homeostasis.

The liver is a key organ in the response to insulin and as it accumulates senescent cells during aging (Hunt et al., 2019), we examined the role of PLA2R1 in regulating marks of liver aging. Telomeres from the livers of old *Pla2r1* KO mice were longer than old WT mice (Figure 1c) and p21 cyclin‐dependent kinase inhibitor levels were lower (Figure S1C). In addition, other hallmarks of chronic liver disease and age‐related liver alterations were examined (Giannini et al., 2005). Old *Pla2r1* KO mice displayed lower levels of blood alkaline phosphatase (ALP; Figure 1d) but showed no altered levels of other blood metabolites examined (Table S1). Fibrosis and steatosis were low in old WT mice; the impact of *Pla2r1* KO was more difficult to evaluate, even though a slight decrease in both was observed (Figure S1D,E). These findings suggest that the loss of *Pla2r1* prevents metabolic dysregulations and attenuates some age‐related hepatic alterations.

**FIGURE 2:**
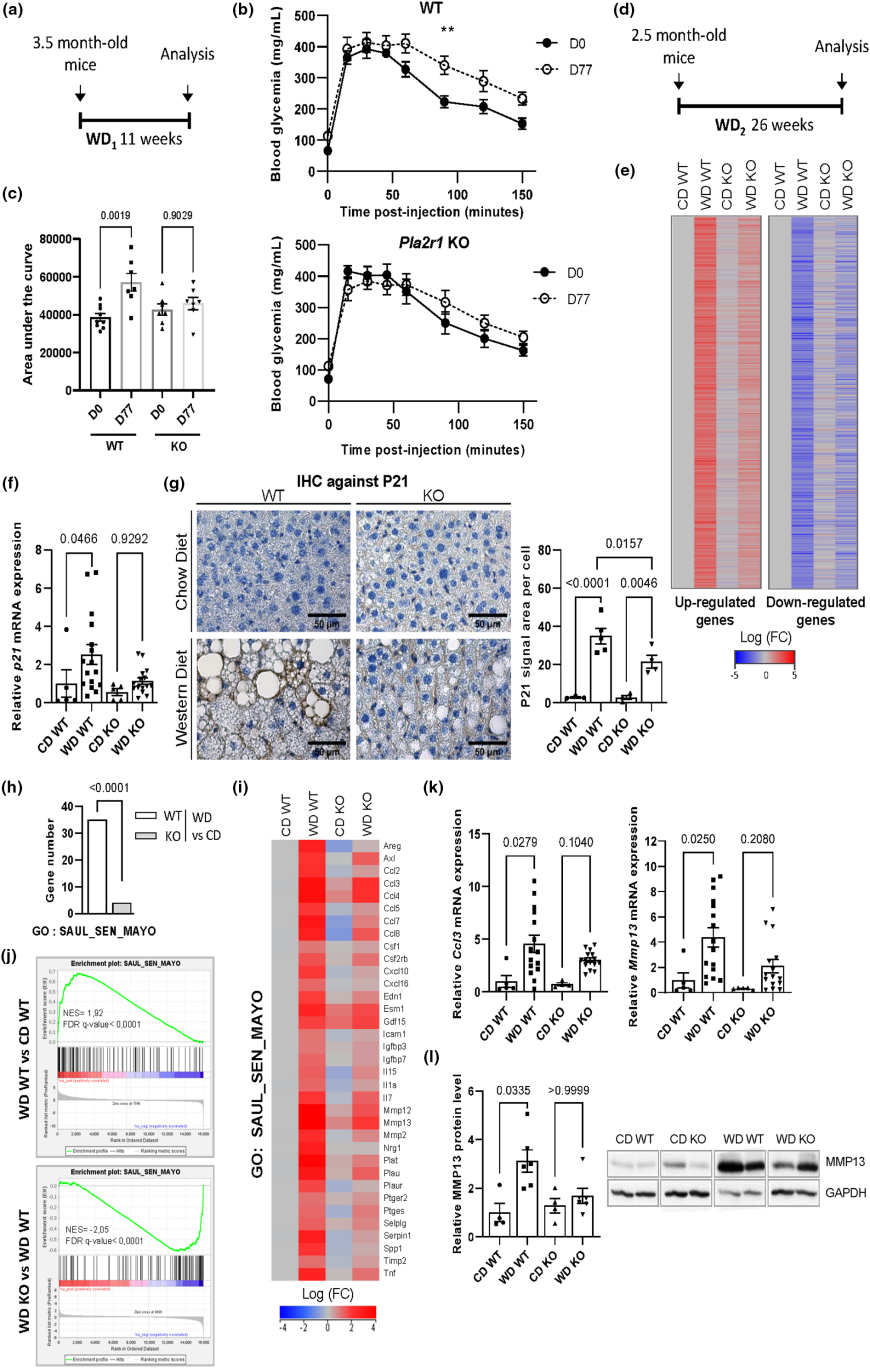
Loss of *Pla2r1* prevents metabolic dysregulation and liver senescence caused by a Western diet. (a) Timeline of the WD1 for the experiments shown in b and c. (b, c) Intraperitoneal glucose tolerance test of wild type (WT; *n* = 7) and *Pla2r1* KO (*n* = 7) mice subjected to a Western diet (WD) at the beginning (D0) and after 11 weeks WD (D77) represented as a curve (b) or histogram showing the area under the curve measured according to Figure 2b (c). Mean ± SEM. (b) Multiple unpaired *t* tests with Welch correction. *p < 0.05, **p < 0.01. (c) ROUT 1% method to identify outliers followed by one‐way ANOVA multiple comparisons test. (d) Timeline of the WD2 for the experiments shown from e to k. (e) Heat‐map showing up‐ and downregulated genes in the liver of WT or Pla2r1 KO mice administered a normal Chow diet (CD) or WD according to transcriptomic microarray analysis in the liver (CD WT = 4; CD KO = 4; WD WT = 4; WD KO = 4). All the genes in CD WT conditions have been normalized to 0 (grey color) to help visualize the variation of gene expression for each gene in the different groups compared with the CD WT reference group. Color scale represents the Log (fold change (FC)). (f, g) Relative *p21* mRNA expression (f) and micrographs of liver sections and quantification of P21 signal area per cell by immunohistochemistry (IHC) (g) of WT and *Pla2r1* KO mice under CD or WD. The number of mice in (f): CD WT = 5; CD KO = 5; WD WT = 16; WD KO = 15; and (g): CD WT = 3; CD KO = 4; WD WT = 5; WD KO = 4, one outlier identified in CD WT group. Mean ± SEM. (f) Kruskal‐Wallis multiple comparison tests (g) and ROUT 1% method to identify outliers followed by one‐way ANOVA multiple comparisons test. (h–j) Gene ontology (GO) “SAUL_SEN_MAYO” analysis shows the number of genes with significant gene expression changes (FC >2; *p* < 0.05) between WD versus CD in WT and *Pla2r1* KO mice (Fisher exact test) (h), heatmap representation (i), and gene set enrichment analysis (CD WT = 4; CD KO = 4; WD WT = 4; WD KO = 4). (k) Relative *Ccl3* and *Mmp13* mRNA expression of WT and *Pla2r1* KO mice under CD or WD (*Ccl3*: CD WT = 5; CD KO = 4; WD WT = 15; WD KO = 16 and *Mmp13*: CD WT = 5; CD KO = 5; WD WT = 15; WD KO = 16). Mean ± SEM. ROUT 1% method to identify outliers them followed by Kruskal–Wallis multiple comparisons test. (l) MMP13 and GAPDH protein levels and relative quantification of MMP13 normalized to GAPDH in the liver of WT and *Pla2r1* KO mice under CD or WD (CD WT = 4; CD KO = 4; WD WT = 6; WD KO = 6). Mean ± SEM. ROUT 1% method to identify outliers followed by the Kruskal‐Wallis multiple comparisons test.

As cellular senescence is involved in metabolic disorders such as NAFLD (Farrell & Larter, 2006; Meijnikman et al., 2021; Papatheodoridi et al., 2020; Schafer et al., 2017) and as these disorders lead to signs of accelerated aging, we wondered whether the loss of *Pla2r1* could impact cellular senescence, glucose regulation, and liver alterations caused by Western diets (WD). To do so, 3.5‐month‐old mice were subjected to a WD for 11 weeks (Figure 2a), and, as expected WT mice grew less tolerant to glucose due to the WD (Figure 2b,c; Chao et al., 2019; Kanuri & Bergheim, 2013). Strikingly, *Pla2r1* KO mice responded in a similar manner to glucose challenge at the beginning (D0) and at the end of the WD (D77; Figure 2b,c), highlighting protection against WD‐induced glucose intolerance. The WD regimen prompted the appearance of metabolic disorders resembling the early stages of NAFLD. To generate more severe liver alterations, such as steatosis and fibrosis, a second more intense WD diet was administered (supplemented with cholesterol, higher amounts of sucrose, and given for 26 weeks) for further experiments (Figure 2d). Transcriptomic analysis performed on the liver revealed that dysregulation of gene expression between WD and control Chow diet (CD) in WT mice were less impacted in the liver of *Pla2r1* KO mice (Figures 2e and S2A–D) in particular 78% of the genes that were significantly downregulated in *Pla2r1* KO WD versus WT WD were increased by the WD regimen in WT (Figure S2E), suggesting that the loss of *Pla2r1* attenuated the response to WD. This treatment led to the accumulation of senescence marks in WT mice: increased p21 cyclin‐dependent kinase inhibitor (Figure 2f,g), enrichment of SASP signatures, including the recently described SAUL_SEN_MAYO (Saul et al., 2022; Figures 2h–j and S3A–C), and some SASP factors at mRNA and protein levels (Figures 2k,l and S3D). Importantly, these increases were largely attenuated in *Pla2r1* KO mice (Figures 2k,l and S3A–D). Beyond the pro‐inflammatory SASP, the broad molecular signature “inflammatory response” supported a global decrease in WD‐induced liver inflammation in *Pla2r1* KO mice (Figure S3E,F). These observations support that *Pla2r1* KO mice are protected from WD‐induced senescence.

### 2.2 Loss of *Pla2r1* decreases the fatty liver phenotype in mice subjected to a Western diet

**FIGURE 3:**
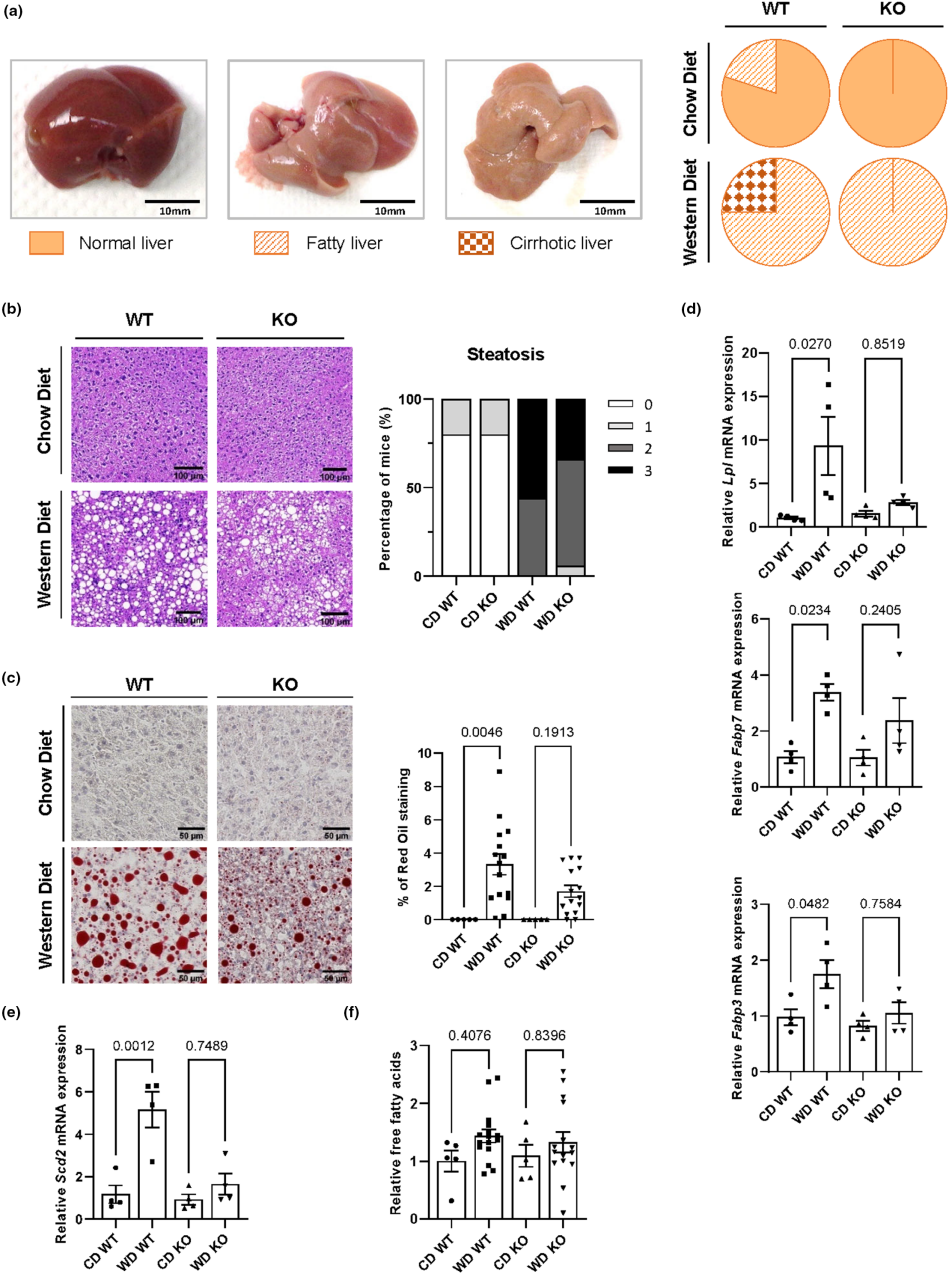
Loss of *Pla2r1* attenuates the fatty liver phenotype in mice subjected to a Western diet. (a) Classification of the livers of wild type (WT) and *Pla2r1* KO mice fed with a normal Chow diet (CD) or a Western diet (WD) according to their macroscopic features. (b, c) Micrographs of liver stained with hematoxylin and eosin (b) and Oil Red O (c) from WT or *Pla2r1* KO mice under CD or WD. (b) Pathological examination of steatosis grade according to Kleiner et al. (2005). (c) Quantification of O Red Oil staining. Mean ± SEM. ROUT 1% method to identify outliers followed by the Kruskal‐Wallis multiple comparisons test. The number of mice in (a, b): CD WT = 5; CD KO = 5; WD WT = 16; WD KO = 15; and (c): same number of mice as for (a, b) excepted for WD WT = 15, one outlier identified. (d, e) Relative expression of genes involved in hepatic uptake of fatty acids *Lpl*, *Fabp7*, and *Fabp3* (d) or in de novo lipogenesis *Scd2* (e) in the liver of WT and *Pla2r1* KO mice given a CD or a WD (CD WT = 4; CD KO = 4; WD WT = 4; WD KO = 4). Data obtained from transcriptomic analysis. Mean ± SEM. Kruskal‐Wallis multiple comparison tests. (f) Relative concentration of free fatty acids in serum of WT and *Pla2r1* KO mice under CD and WD (CD WT = 5; CD KO = 5; WD WT = 16; WD KO = 15). Mean ± SEM. One‐way ANOVA multiple comparisons test.

We next explored the role of PLA2R1 in steatosis, one of the main liver alterations associated with NAFLD development (Farrell & Larter, 2006; Nassir et al., 2015). Steatosis is defined as abnormal lipid accumulation in hepatocytes, which impacts glucose homeostasis (Klover & Mooney, 2004; Li et al., 2020). The proportion of fatty and cirrhotic livers observed in mice given a WD was higher in WT mice compared to *Pla2r1* KO mice, according to macroscopic observations of the liver (Figure 3a). Consistently, H&E staining of the livers confirmed that *Pla2r1* KO mice displayed less severe forms of steatosis (Figure 3b), and Oil Red O staining of the lipid droplets highlighted the reduction of lipid accumulation in the liver of these mice compared with WT mice (Figure 3c).

Hepatic lipid accumulation may be induced by four separate mechanisms: (i) increased hepatic uptake of fatty acids (FA), (ii) increased hepatic de novo fatty acid synthesis, (iii) decreased hepatic beta‐oxidation, and/or (iv) decreased hepatic lipid export (Ipsen et al., 2018). We, therefore, explored the expression levels of genes involved in these different pathways. As shown in Figure 3d, in WT mice, the WD mainly increased the expression in the liver of *Lpl*, *Fabp7*, and *Fapb3*, genes involved in the hydrolysis of triglycerides (TG), and FA uptake. This induction was largely reversed in *Pla2r1* KO mice. Similar trends were observed with the expression of *Fabp4* and *Cd36*, other members of FA uptake in *Pla2r1* KO mice (Figure S4A). De novo lipogenesis might also be impacted as *Scd2* upregulation by WD in WT mice was lost in *Pla2r1* KO mice (Figure 3e), though other genes of this pathway were not differentially regulated by the WD (Figure S4B). Genes involved in regulating β‐oxidation and lipid export were not significantly impacted by the WD and/or PLA2R1 (Figure S4C,D). Therefore, it seems that the loss of *Pla2r1* could decrease lipid accumulation mainly through a decrease in TG hydrolysis and hepatic uptake of fatty acids, and also maybe by reducing hepatic de novo lipogenesis, consequently leading to less severe hepatic steatosis. As levels of circulating‐free FA and TG were not impacted by *Pla2r1* KO mice upon WD (Figure 3f and Table S2), the phenotypes might be more dependent on hepatic alterations than adipose tissue alterations, which should have impacted circulating‐free FA and TG. Of note, induction of ALP and ALAT by the WD in WT mice was attenuated in the *Pla2r1* KO mice, also suggesting a more liver‐specific phenotype (Table S2).

Therefore, the loss of *Pla2r1* might decrease lipid accumulation mainly through a decrease in the liver of the synthesis and uptake of lipids to hepatocytic droplets, leading to less severe hepatic steatosis.

### 2.3 Liver fibrosis is attenuated in *Pla2r1* knockout mice subjected to a Western diet

**FIGURE 4:**
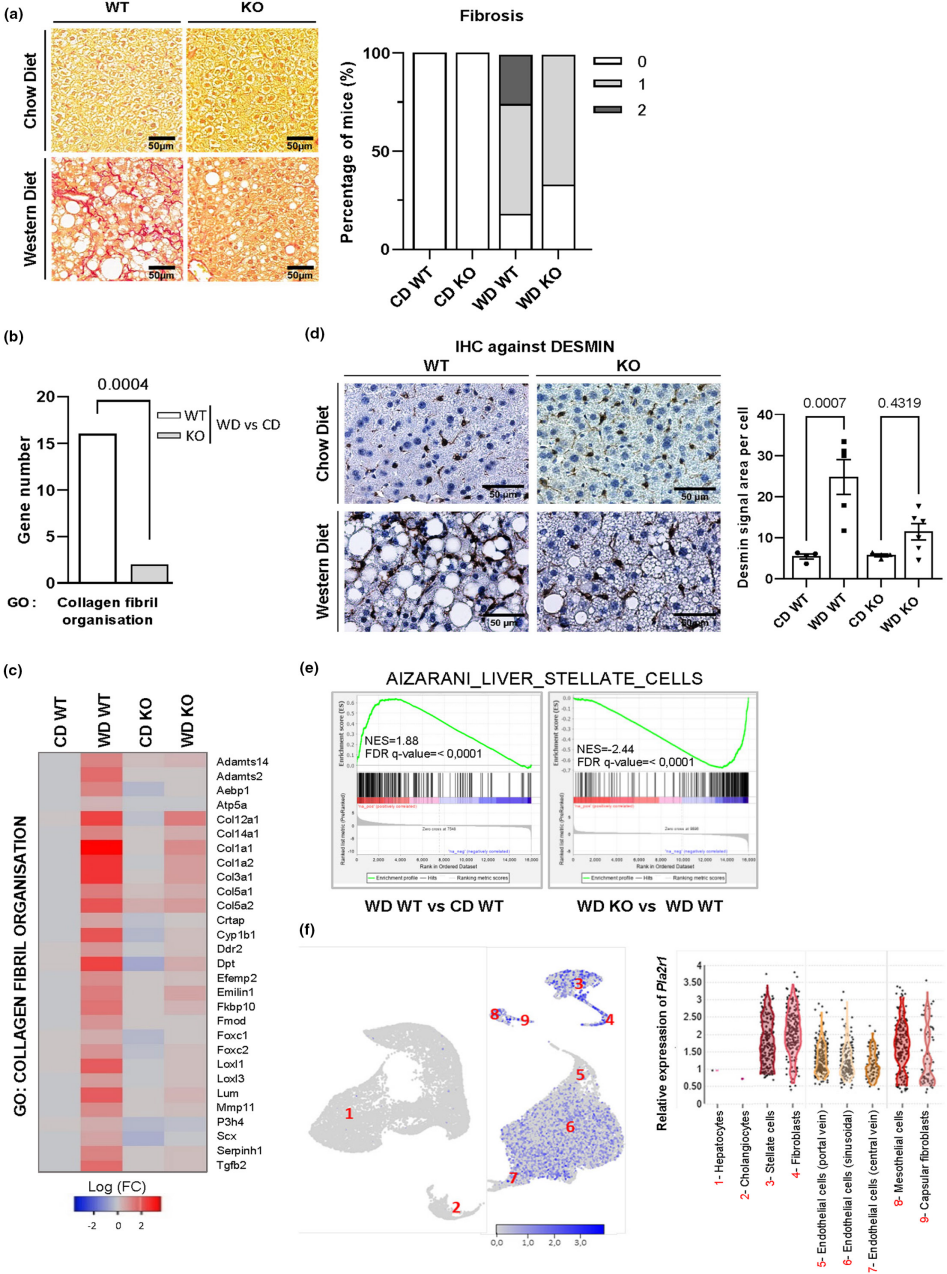
Livers from *Pla2r1* knockout mice fed a Western diet display less fibrosis. (a) Micrographs of liver stained with Sirius Red from wild type 3(WT) and *Pla2r1* KO mice given a normal Chow diet (CD) or a Western diet (WD) and pathological examination of fibrosis grade according to Kleiner et al., 2005 (CD WT = 5; CD KO = 5; WD WT = 16; CD KO = 15). (b, c) GO analysis shows the number of the genes (FC >2; *p* < 0.05) involved in collagen fibril organization and the associated with the heatmap between WD and CD in WT and *Pla2r1* KO mice. Fisher exact test. (d) Micrographs of liver sections and quantification of DESMIN signal area per cells by immunohistochemistry (IHC) from WT and *Pla2r1* KO mice fed a CD or WD (CD WT = 4; CD KO = 4; WD WT = 5; WD KO = 6). Mean ± SEM. One‐way ANOVA multiple comparisons test. (e) Gene set enrichment analysis shows enrichment of gene sets related to Aizarani liver stellate cells from the liver of WT or *Pla2r1* KO mice fed a CD or WD according to transcriptomic analysis (CD WT = 4; CD KO = 4; WD WT = 4; WD KO = 4). (f) Relative *Pla2r1* mRNA level in different liver cell types from public scRNA‐seq of the murine liver (https://www.livercellatlas.org/).

It is well known that fibrosis, characterized by abnormal collagen deposition, defines more severe forms of NAFLD (Bataller & Brenner, 2005; Farrell & Larter, 2006). To quantify collagen deposition, liver tissue sections were stained with Sirius Red and examined to grade fibrosis (Dees et al., 2021). WT mice showed more severe forms of fibrosis than *Pla2r1* KO mice in response to the WD (Figure 4a). Gene Ontology analysis performed on transcriptomic data revealed an enrichment in the “collagen fibril organization” signature for upregulated genes in WT mice subjected to a WD, which was largely reverted in *Pla2r1* KO mice according to heatmap and GSEA analysis (Figures 4b,c and S5A). Expression of *Col1a1*, a major component of collagen, and of *Tgfb1* an important promoter of fibrosis (Kim et al., 2018; Walton et al., 2017) was also stronger following administration of the WD in WT mice compared with *Pla2r1* KO mice (Figure S5B,C), further supporting that *Pla2r1* KO mice were protected from liver fibrosis.

Hepatic stellate cells (HSCs) are a major mediator of liver fibrosis as they migrate to the site of damage, proliferate, and produce extracellular matrix, such as collagen (Bataller & Brenner, 2005; Moreira, 2007). As expected, the quantity of DESMIN‐positive cells, a marker of HSCs, and the enrichment in the liver stellate molecular signature indicated an increase in the number of HSCs in WT mice given a WD. This increase was largely reversed in *Pla2r1* KO mice subjected to the same diet (Figure 4d,e). Through single‐cell RNA‐sequencing (scRNA‐seq), we ruled out a direct effect of PLA2R1 on hepatocytes, which did not express *Pla2r1*, unlike stellar cells (Figure 4f).

Taken together, these data suggest that the loss of *Pla2r1* protects the liver from fibrosis arising from a WD.

## 3 DISCUSSION

In this study, we unraveled a role for *Pla2r1* in contributing to age‐/WD‐related hepatic alterations and induction of cellular senescence. Indeed, we demonstrated that *Pla2r1* was directly involved in decreasing the ability of aged/WD‐fed mice to regulate their glucose and fat metabolism, leading to disorders such as hepatic steatosis, fibrosis, and cirrhosis.

Altered glucose metabolism and immunosenescence increase with aging, and both participate in a wide spectrum of diseases including diabetes, cardiovascular diseases, and infectious diseases (Cox et al., 2020; Goronzy & Weyand, 2013; Kalyani & Egan, 2013; Risk et al., 2018). Here, we observed that aged *Pla2r1* KO mice display fewer signs of aging: to some extent lower immunosenescence and a clear ability to regulate their glucose metabolism when tolerance tests were performed. Strikingly, the loss of *Pla2r1* also protected mice from an accelerated dysfunctional glucose metabolism induced by Western diets, further confirming a major role for *Pla2r1* in regulating glucose homeostasis. As far as we know, *Pla2r1* has never been implicated in this function before. In line with these results and a deleterious effect of *Pla2r1*, it was shown to promote features of progeroid syndromes (Griveau et al., 2018), marks of chronic obstructive pulmonary diseases (COPD; Beaulieu et al., 2021) and to increase sensitivity to endotoxic shock (Hanasaki et al., 1997). Nevertheless, in some cases, *Pla2r1* exerts beneficial effects as its deletion increases inflammation and allergic sensitization in an asthma model (Nolin et al., 2016), decreases survival rate due to the increase in cardiac rupture after myocardial infarction (Mishina et al., 2014), and decreases tumor initiation during aging (Huna et al., 2021) or after carcinogenic treatment (Vindrieux et al., 2013).

In some of the reported pathophysiological functions of *Pla2r1*, its effects are functionally linked to its pro‐senescent functions. For instance, PLA2R1‐induced senescence promotes progeroid marks (Griveau et al., 2018), COPD features (Beaulieu et al., 2021), or impairment of tumorigenesis (Huna et al., 2021; Vindrieux et al., 2013). Whether the other effects of *Pla2r1* rely on its pro‐senescence function has not been investigated but endotoxic shock (Merdji et al., 2021), asthma (Barbé‐tuana et al., 2021), or myocardial infarction (Mehdizadeh et al., 2022) can impact cellular senescence. In our study, the loss of *Pla2r1* was associated with decreased levels of senescence marks and a better ability to regulate glucose metabolism, as well as a reduction in liver steatosis and fibrosis, which is consistent with the fact that senescent cell accumulation can promote these alterations (Ogrodnik et al., 2017; Ogrodnik & Jurk, 2017). Nevertheless, cellular senescence also exerts beneficial effects, for instance, by blocking tumorigenesis or promoting wound healing (Demaria et al., 2014; He & Sharpless, 2017).

Whether the beneficial or deleterious effects of *Pla2r1* on health are directly correlated with the beneficial or deleterious effects of senescent cells is an open question. For instance, *Pla2r1* promoted collagen deposition in the liver during a Western diet in our study and was shown to do so in mouse lungs when *Pla2r1* is overexpressed (Beaulieu et al., 2021) or in the heart after myocardial infarction (Mishina et al., 2014). However, in the first two cases, *Pla2r1* is associated with fibrosis, whereas in the latter, it is associated with healing as a prevention of cardiac rupture (Mishina et al., 2014). Even though senescence was not investigated in the Mishina et al. study, cellular senescence could contribute to heart regeneration (Feng et al., 2019). This might reveal a multifunctional role for *Pla2r1* with negative or positive effects on fibrosis depending on the context (Aghali et al., 2022; Krizhanovsky et al., 2008; Mehdizadeh et al., 2022). As senescence participates in healing (Demaria et al., 2014) and as fibrosis can be considered to be a permanent dysfunctional healing process (Diegelmann & Evans, 2004), *Pla2r1*, as a pro‐senescence factor, might promote healing or fibrosis depending on the context and on its transient or permanent activation. The pro‐ or antifibrotic role of senescent HSC on the regulation of liver fibrosis is still under debate (Krizhanovsky et al., 2008). Nevertheless, a recent article demonstrated that elimination of senescent HSCs by generating CAR‐T cells directed against PLAUR, a cell surface marker of senescent cells, decreases liver fibrosis upon a Western diet (Amor et al., 2020). As *Pla2r1* was herein expressed by HSCs and not hepatocytes according to scRNA‐seq, as *Pla2r1* KO display decreased PLAUR levels during WD, we can speculate that liver phenotypes observed in *Pla2r1* KO mice rely at least in part on decreased cellular senescence of HSCs and their paracrine function including on hepatocytes.

NAFLD is characterized by a wide range of alterations from a simple fatty liver and steatosis to NASH, characterized by fibrosis, inflammation, insulino‐resistance and cirrhosis, or even to hepatocellular carcinoma in the most severe forms of the pathology (Anstee et al., 2019; Bessone et al., 2019; Younossi et al., 2018). Our results identify a potential role for *Pla2r1* in promoting several of the alterations associated with NASH including cellular senescence, steatosis, and fibrosis and generating a histopathological score compiling steatosis, ballooning, and fibrosis further supports the findings (Figure S6). This work paves the way for future investigations focusing on the mechanisms by which PLA2R1 regulates liver biology and whether it, or the pathway it regulates, can constitute promising therapeutic targets.

Overall, our results support that PLA2R1 regulates some age‐related phenotypes during physiological aging or following a Western diet and opens new perspectives to improve our understanding of the biology of aging as well as a new mechanism contributing to metabolic disorders.

## 4 MATERIALS AND METHODS

### 4.1 Animals and in vivo protocols

C57BL/6 *Pla2r1* wild‐type (WT) and *Pla2r1* KO littermate mice were used for experiments. *Pla2r1* KO mice were genotyped as previously described (Hanasaki et al., 1997). For Western diet experiments, two different diets were administered: (i) 3.5‐month‐old male mice were fed with 21.2% fat, 48.5% carbohydrates, and 17.3% proteins which included 341.46 g of sucrose/kg and 0.15 g of cholesterol/kg (TD.88137, Envigo) for the Intraperitoneal Glucose Tolerance Test (IPGTT), (ii) 2.5‐month‐old mice were either fed a normal Chow diet (TD‐08485, Envigo) and given normal tap water, or subjected to a Western diet with 21.2% fat, 48.5%, carbohydrate, and 17.3% proteins which included 405.36 g of sucrose/kg and 1.25 g of cholesterol/kg (TD.120528, Envigo) and were hydrated with water containing 23.1 g/L D‐Glucose (G8270, Sigma) and 18.9 g/L D‐Fructose (F0127, Sigma) for 26 weeks for all other experiments. Animals were maintained in a specific pathogen‐free (SPF) animal facility on the P‐PAC platform at the Cancer Research Center of Lyon (CRCL). All of the experiments were performed in accordance with the regulations for animals used for scientific purposes governed by the European Directive 2010/63/EU. Protocols were validated by the local Animal Ethics Evaluation Committee (CECCAPP:C2EA‐15) and authorized by the French Ministry of Education and Research.

### 4.2 Intraperitoneal glucose tolerance test (IPGTT), intraperitoneal insulin tolerance test (IPITT), and insulin production

To test glucose tolerance and measure insulin production, mice were deprived of food for 16 h before receiving an intraperitoneal administration of 2.5 or 2 g/kg glucose (G8270, Sigma) for males and females, respectively. Their blood glucose was measured using a blood glucometer at the tip of their tails (FreeStyle PAPILLON Vision, Abbott), before (0 min) and at the indicated times and the insulin was measured (ALPCO‐80‐INSMS‐E01 from Eurobio) before (0 min) and at 15 min after glucose injection. To test insulin tolerance, mice were deprived of food for 6 h before receiving an intraperitoneal administration of 1 U/kg insulin (ACTRAPID© 100 UI/mL, NOVO NORDISK), and their blood glucose was measured before (0 min) and at the indicated times using the protocol described below.

### 4.3 Blood metabolites and free fatty acid (FFA) measurements

Bloods were collected in a tube with no anticoagulant and let it clot at room temperature for 30 min. Bloods were then centrifuged at 2500 × *g* for 20 min. The serum supernatants were stored at −80°C.

Metabolites analyses were performed on the ARCHITECT c 16000 Systems (Abbott laboratory). For free FA measurement, free FA was measured in duplicate according to the manufacturer's recommendations (Cell Biolabs, # STA‐618).

### 4.4 Immunohistochemistry, hematoxylin and eosin, Sirius red, and oil red O staining of the liver

Livers were collected and fixed in 10% formalin for 24 h and then in 70% ethanol before paraffin embedding. Paraffin‐embedded murine tissues were serially sectioned at a 3–4‐μm thickness (for immunohistochemistry [IHC], hematoxylin and eosin [H&E], and Sirius red staining) de‐waxed and hydrated.

All IHC assays were performed following peroxidase inhibition with H_2_O_2_ and antigen retrieval with citrate buffer. For immunohistological DESMIN staining, the primary antibody diluted 1:200 (ab15200, Abcam) was incubated overnight, before adding the rabbit secondary antibody (BA‐1100, Vector laboratories) diluted 1:200 for 1 h. For p21 staining, the manufacturer's recommendations for the mouse‐on‐mouse immunodetection kit (BMK‐2202, Vector Laboratories) were followed and p21 antibody was used at a 1:100 dilution (sc‐271,610, Santa Cruz). IHC staining was visualized using 3,3′‐diaminobenzidine (DAB Kit, Vector Laboratories) and sections were counterstained with Mayer's hematoxylin (C0303, Diapath, MicromMicrotech) before dehydration and mounting. Quantification of signal area per cell was performed automatically using ImageJ software.

For Sirius Red staining, nuclei were first stained with Weigert's hematoxylin. Sections were stained with 0.1% Picro‐sirius red in saturated aqueous picric acid solution (Direct Red 80, CI 35782, D0303, Sigma‐Aldrich and P6744‐1GA, Sigma‐Aldrich) for 1 h and washed in acidified water (0.5% of acetic acid) to stain type I and III collagen. Finally, slices were dehydrated before mounting. The distribution of fibrous deposits, their density, and their intensity were assessed and compared with normal collagen deposition in hepatic lobules. The fibrotic score was defined as described in Kleiner et al: Grade 0: only normal collagen deposit observed, for instance in hepatic portal space; Grade 1: increased deposit in perisinusoidal or portal/periportal; Grade 2: increased deposit in perisinusoidal and portal/periportal.

For H&E, slides were first immersed in Mayer's hematoxylin and then immersed in Eosin G 1% dye (C0363, Diapath, MicromMicrotech) for 2 min and dehydrated before mounting.

For Oil Red O staining, livers were collected, snap‐frozen, and sectioned at an 8‐μm thickness. Slices were then stained with freshly prepared Oil Red O (0.3% of CI26125, Sigma) working solution for 15 min and rinsed with 60% isopropanol. Nuclei were then slightly stained with Mayer's hematoxylin (5 dips) before aqueous mounting. Steatosis was assessed by quantification of hepatocytes having optically‐empty vacuoles in their cytoplasm. To be diagnosed as having a triglyceride origin, vacuoles had to be well delineated, and compressing hepatocyte nuclei towards cell periphery. Grade 0: < 5%; Grade 1: > 5–33%, Grade 2: > 33–66, and Grade 3: > 66%.

Images were acquired under a light microscope (Axioscan Z1, Zeiss) before analysis with ImageJ software.

### 4.5 Immunophenotyping

#### 4.5.1 Cell isolation

Spleen and lymph nodes were harvested, mechanically disrupted, and filtered through a sterile 100‐μm nylon mesh filter (BD Biosciences). Bone marrow cells were isolated from the tibias and femurs using an 18‐gauge needle containing complete RPMI, and then single‐cell suspensions were prepared after RBC lysis (ACK).

#### 4.5.2 Flow cytometry

Cells were stained for 30 min at 4 °C with the appropriate mixture of mAbs diluted in staining buffer (PBS supplemented with 1% FCS (Life Technologies) and 0.09% NaN3 (Sigma‐Aldrich, Saint Quentin‐Fallavier, France)). The following Abs (clones) were used: anti‐mouse CD45 (30‐F11); anti‐mouse CD3e (145‐2C11); anti‐mouse CD4 (RM4.5); anti‐mouse CD8 (53–6.7); anti‐mouse CD44 FITC (IM7); anti‐mouse CD62L (MEL‐14); anti‐mouse PD1 (29F.1A12); anti‐mouse CD34 (RAM34); all BD PharMingen. Anti‐mouse KLRG1 (2F1/KLRG1); anti‐mouse B220 (RA3–6B2); anti‐mouse Sca‐1 (D7), anti‐mouse CD117 (2B8); anti‐mouse CD11b (M1/70); anti‐mouse Ter119 (Ter119), anti‐mouse Gr‐1 (RB6–8C5), anti‐mouse CD5 (53–7.3); anti‐mouse CD135 (A2F10); anti‐mouse Fcγ R III (93); anti‐mouse CD127 (A7R34); all Biolegend. Live Dead Aqua 405 nm (Invitrogen) was used to exclude dead cells from the analysis.

For analysis of T and B cells: Viable lymphocytes were addressed by gating in the SSC‐A/FSC‐A plot. Then, singlets were gated in an FSC‐A/FSC‐H plot. B cells were gated in a B220/CD45 plot. CD3+ T cells were gated in a CD3/CD45 plot. From CD3+ T cells, CD4+ and CD8+ T cells were gated in CD4/CD8 plots. CD4 and CD8 T cells were further categorized into memory and naive phenotypes based on CD62L (L‐selectin) and CD44 expression: Naive cells were identified as CD44lowCD62Lhigh cells, and memory was identified as CD44high population. Among CD4+ or CD8+ cells, KLRG1+ cells and PD1+ were, respectively, gated in a CD44/KLRG1 plot and CD44/PD1 plot.

For analysis of hematopoietic stem and progenitor cells (HSPC): Dead cells were excluded using Live Dead Aqua. Then, singlets were gated in an FSC‐A/FSC‐H plot. HSPC cells were gated by lineage‐negative cells, followed by gating for Sca‐1 and c‐Kit (CD117). HSPC subsets were next identified based on the expression of CD34 and Flt‐3 (CD135) and each subset were characterized with the following phenotypes: LT‐HSC as Lin − CD117 + Sca‐1 + CD34 − Flt3−; ST‐HSC as Lin − CD117 + Sca‐1+ CD34 + Flt3−; and MPP as Lin − CD117 + Sca‐1 + CD34+/−Flt3+/−.

For measurements of IFNγ production, 1.10^6^ splenocytes were cultured for 4 h with plate‐bound anti‐CD3 Ab (145‐2C11, 10 μg/mL; BD Biosciences) and soluble anti‐CD28 Ab (37.51, 1 μg/mL; BD Biosciences) or with Cell Activation Cocktail (423301, Biolegend) in the presence of GolgiStop (BD Biosciences). To perform intracellular staining, cells were fixed and permeabilized using CytoFix/CytoPerm (BD Pharmingen). The following Abs (clones) were used for intracellular staining: IFN‐γ (XMG1.2), TNFα (MP6‐XT22).

Flow cytometry analyses were performed on a Becton Dickinson FACS Fortessa LSRII and analyzed with the FlowJo software (TreeStar, Asland, OR, USA).

### 4.6 Telomere length measurement

Genomic DNA was extracted from the liver of mice using the DNeasy Kit (QIAGEN, France) and quantified with Nanodrop. 30 ng of gDNA was used as a template for quantitative PCR (qPCR) and mixed with primers of telomere (Cawthon, 2002) and 36B4 (acidic ribosomal phosphoprotein PO, a single‐copy gene for normalization) at a final concentration of 300 nM and SYBR™ Green PCR Master Mix (ThermoFisher Scientific). Primer sequences are described in the Table S3. Each reaction was performed in triplicate. Signal detection and analysis of the results were made using the QuantStudio Real‐Time PCR Software (Applied Biosystems).

### 4.7 RNA extraction, reverse transcription, and real‐time quantitative PCR

About 50 mg of liver tissue was shredded using a tissue homogenizer Precellys (P000062‐PEVO0‐A) and Precellys Lysing Kit (P000912‐LYSK0) in 500 μL of Nucleozol (740404.200, Macherey Nagel) and RNA was then extracted according to the manufacturer's recommendations.

Synthesis of cDNA was performed using a Maxima First cDNA Synthesis Kit (K1641, ThermoFisher Scientific) from 1 μg of RNA. Generated cDNA (50 ng/μL) was used as a template for qPCR run and mixed with primers (200 nM) for the gene of interest and SYBR™ Green PCR Master Mix (ThermoFisher Scientific). qPCR analyses were carried out with the FX96 Thermocycler (Biorad, Hercules, US). Relative mRNA levels were calculated using the Comparative Ct (2^−ΔΔCT^) method. mRNA levels of 4 (Actb/Gapdh/Tbp/Rplp) housekeeping genes were used for normalization. Primer sequences targeting genes of interest and housekeeping genes are listed in the Table S3.

### 4.8 Immunoblots

For immunoblot experiments, frozen livers were lysed and sonicated in RIPA buffer. After protein quantification, 30 μg of proteins were loaded and resolved by SDS‐PAGE electrophoresis and transferred to nitrocellulose membranes (Bio‐Rad). Membranes were blocked with TBS‐Tween/Milk 5% for 1 h and incubated at 4°C with primary antibodies overnight. The following antibodies and their concentration were used: PLAUR (1/500e, sc‐376494, Santacruz), MMP13 (1/500e, MA5‐14238, Invitrogen), and GAPDH (1/1000e, sc‐32233, Santacruz). Membranes were then incubated with a secondary antibody mouse (715‐035‐150, Interchim) or rabbit (715‐035‐152, Interchim) 1 h at room temperature. Detection was performed using an ECL kit either from Biorad (#1705062) or from ThermoScientific (#32201).

### 4.9 Transcriptomic analysis

Transcriptome analysis of liver tissue from WT or *Pla2r1* KO mice fed a Chow diet or Western diet was performed using the Agilent microarray technology (Whole Mouse Genome Microarrays 4 × 44K v2, G4846A, Agilent Technologies). Briefly, total RNA was extracted from tissue using the Precellys Lysing Kit and Nucleospin RNA kit (R740933.1, Macherey‐Nagel). RNA concentration and quality (RIN index) were controlled using the 2200 TapeStation system (Agilent technologies). Starting from 100 ng of total RNA, cDNAs were synthesized and used to produce Cy3‐labeled cRNA using the Agilent one‐color labeling kit (Quick Amp Labeling Kit, one‐color, Agilent). Then, 1650 ng of Cy3‐labeled cRNA was hybridized on 4X44K arrays for 17 h at 65°C. Microarrays were washed and scanned with an Agilent DNA microarray scanner G2565CA (Agilent Technologies). Fluorescent signals were extracted and normalized with Feature Extraction Software Version 10.5.1.1 (Agilent Technologies) and transferred to Genespring GX 12.6 software (Agilent Technologies) for data processing and data mining. Data were normalized in Genespring using the 75th percentile method and probes were filtered using a probe set filter to remove probes with raw signals below 10 in all of the conditions tested. Differentially expressed genes (DEGs) were defined by volcano plot analysis using moderated *t* test *p*‐value <0.05 with a fold change cutoff of 2‐fold for up‐ and downregulation. For data visualization, hierarchical clustering was performed with the Euclidian metric and complete linkage method. Preranked GSEA was performed on ranked lists of gene expression ratio, between average expression in the two conditions studied using the GSEA v2.0.13 software with default parameters. All gene set files for this analysis were obtained from the GSEA website (www.broadinstitute.org/gsea/).

### 4.10 Statistical analysis

To identify possible outliers and perform statistical analyses, the ROUT method of 1% was applied. This information is indicated in the figure legend when the test identified outliers and in this case, the number of mice corresponds to the number of mice after removing outliers. A Shapiro–Wilk normality test was then used for all the experiments. For the analysis of the two groups, an unpaired *t* test or Mann–Whitney test was used, depending on the normality test results. For analysis on more than two groups, ANOVA or Kruskal–Wallis test was used, depending on the normality test results. All the statistical analyses were performed using GraphPad Prism 9.

## AUTHOR CONTRIBUTIONS

D.V., D.G., S.J., A.G., J.R., and A.M. managed mice cohort, follow‐up, tissue processing, tissue staining and analysis. S.D. performed immunophenotyping and E.M. performed telomere length analysis. L.P. analyzed blood samples. J.R. measured insulin level. A.T. performed pathological analysis of the liver. J.M.F and A.M. performed transcriptomic experiments and analysis. A.M., J.M., R.P., S.A., P.B., J.R., A.T., D.V., and D.B. designed the experiments and the results were analyzed by all the co‐authors. D.B. and D.V. supervised the work. D.B. and A.M. wrote the manuscript with input from all the co‐authors.

## CONFLICT OF INTEREST STATEMENT

The authors have declared that no conflict of interest exists.

## Supporting information


Appendix S1.

## Data Availability

The data that support the findings of this study are available from the corresponding author upon reasonable request.
