# Supplementary material for: Loss of Pla2r1 decreases cellular senescence and age‐related alterations caused by aging and Western diets
Source: Aging Cell. 2023 Sep 4;22(11):e13971. doi: 10.1111/acel.13971 (PMC10652324; doi:10.1111/acel.13971)
Supplement: Supplementary file 1 — Appendix S1. [file ACEL-22-e13971-s001.pdf]

Supplementary Figure 1

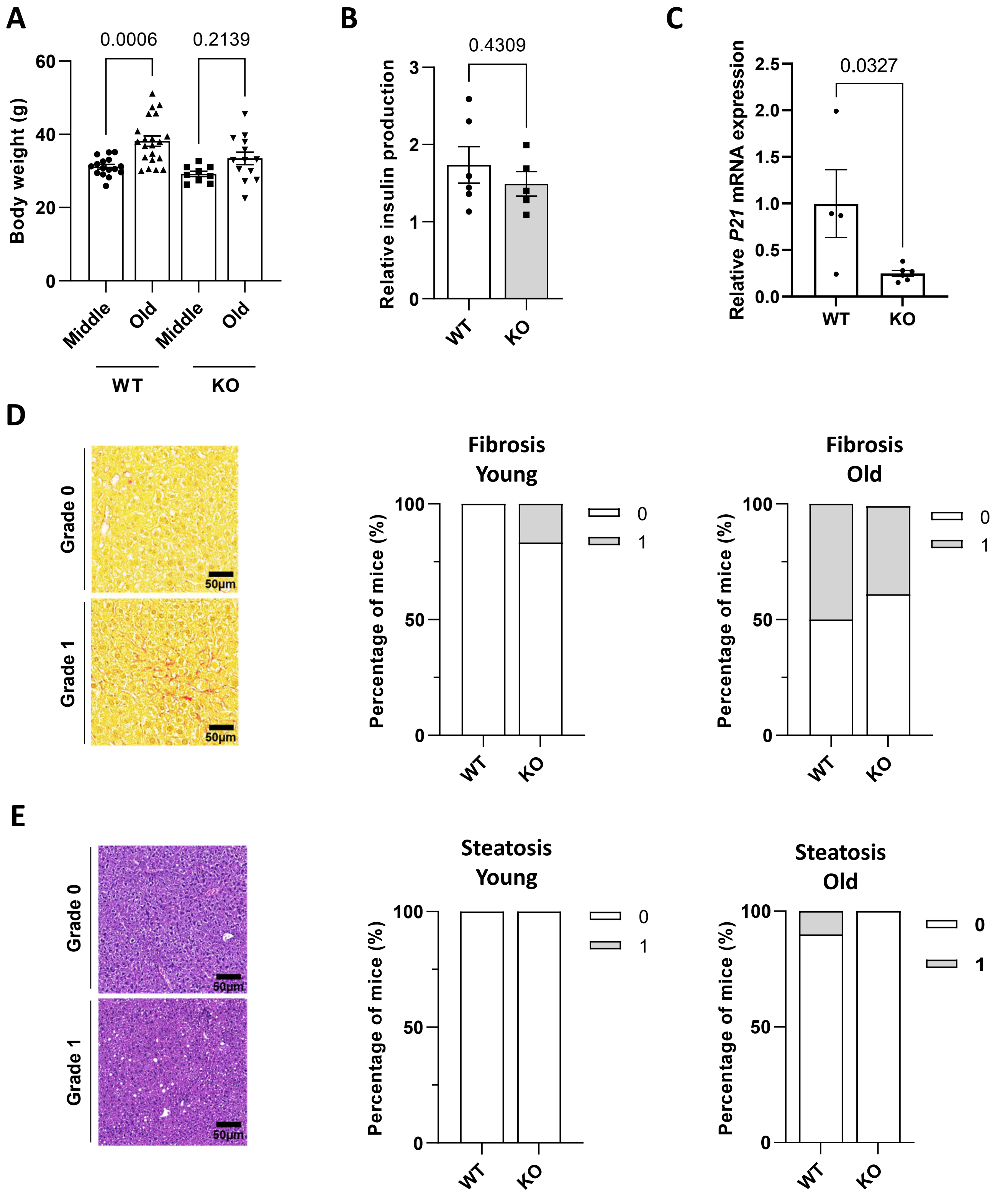

Figure S1 (A) Body weight of wild type (WT) and *Pla2r1* KO at middle age (45 +/- 5 weeks) and at old age (90 +/- 5 weeks). The number of mice at middle age: WT = 16, *Pla2r1* KO = 9 and at old age: WT = 21 and *Pla2r1* KO = 13. Mean +/- SEM. ROUT 1% method to identify outliers followed by one-way ANOVA multiple comparisons test. (B) Relative insulin production of 20-month-old WT (n = 6) and *Pla2r1* KO (n = 5) female mice measured at 15 min vs 0 min after glucose injection. Mean +/- SEM. Unpaired two-tailed Student t-test. (C) Relative *p21* mRNA expression in liver of 21-month-old WT and *Pla2r1* KO mice (D-E) Representative images and pathological examination of fibrosis grade (D) and steatosis grade (E) of livers from 6-month-old (young) WT (n = 6) and *Pla2r1* KO (n = 6) and 21-month-old (old) WT (n = 10) and *Pla2r1* KO (n = 21) mice.

Supplementary Figure 2

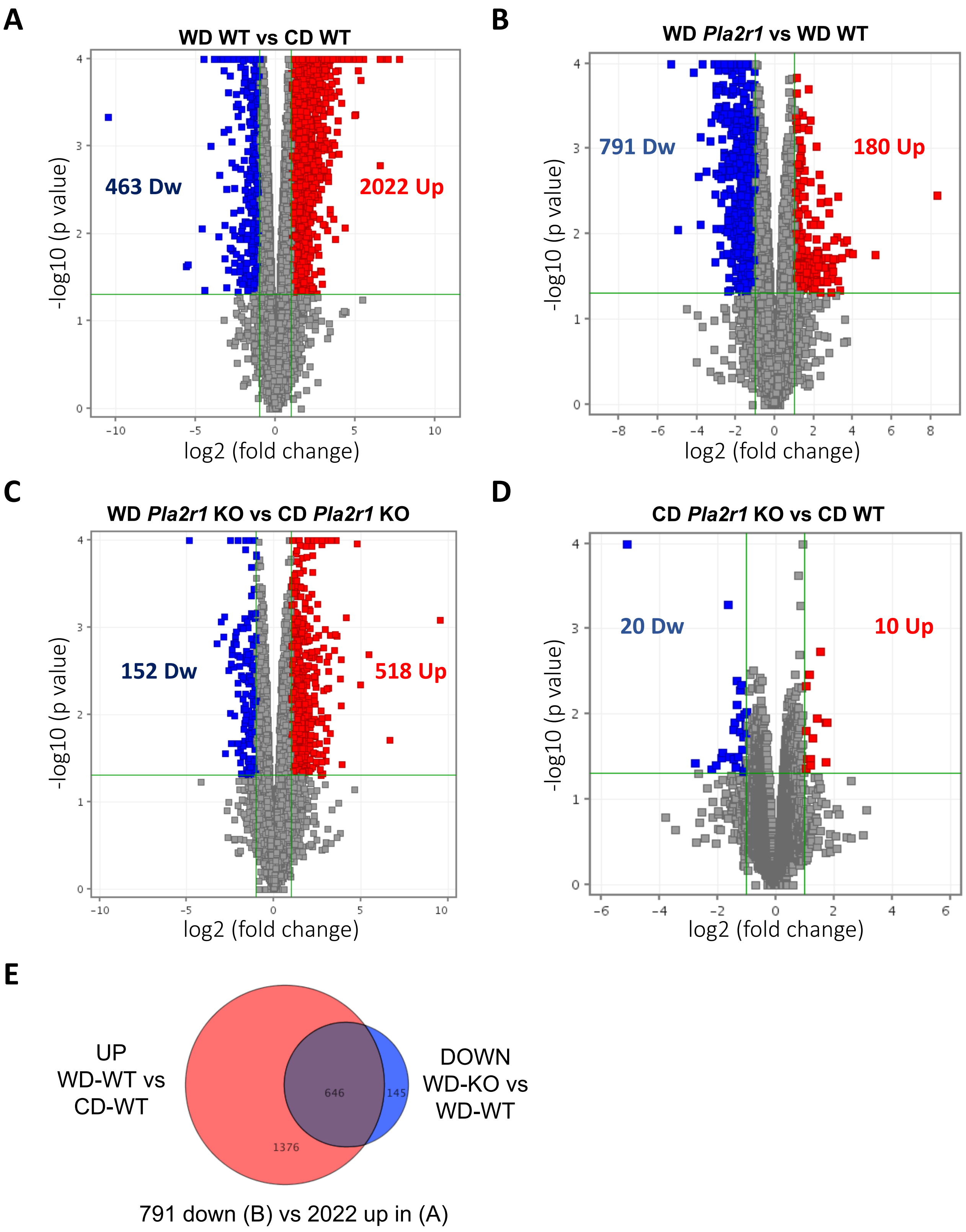

Figure S2 Analysis of transcriptomic data generated from livers of wild type (WT) and *Pla2r1* KO mice fed by a Chow diet (CD) or a Western diet (WD). (A-D) Volcano plots showing the number of differentially expressed genes (FC > 2; p < 0.05) between WD WT and CD WT (A), WD *Pla2r1* KO and WD WT (B), WD and CD *Pla2r1* KO (C) and CD *Pla2r1* KO and CD WT (D). (E) Venn diagram between the up-regulated genes in the liver between WD WT vs CD WT mice and down-regulated genes between WD *Pla2r1* KO vs WD WT mice (CD WT = 4; CD KO = 4; WD WT = 4; WD KO = 4).

Supplementary Figure 3

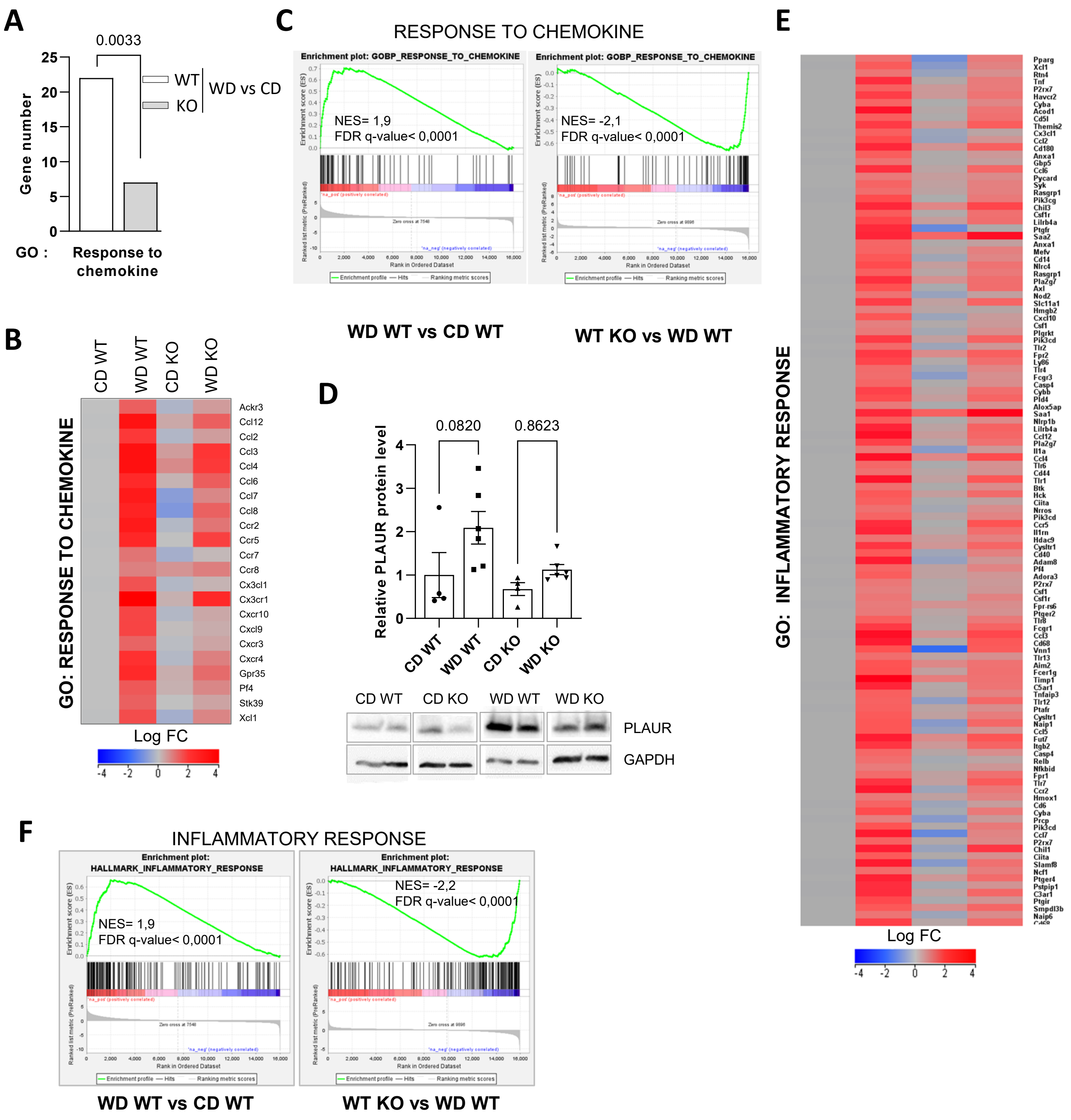

Figure S3 (A-C) Analysis of the Gene ontology (GO) “Response to chemokine” from transcriptomes obtained from livers of wild type (WT) and *Pla2r1* KO mice fed by a Chow diet (CD) or a Western diet (WD). (A) Number of genes with significant gene expression changes (FC > 2; p < 0.05) between WD vs CD in WT and in *Pla2r1* KO mice (Fisher exact test). (B) the heat-map representation and (C) Gene set enrichment analysis (GSEA). (CD WT = 4; CD KO = 4; WD WT = 4; WD KO = 4). (D) Representative immunoblots, using liver samples, against PLAUR, and GAPDH used for normalization, and histograms showing quantification. (CD WT = 4; CD KO = 4; WD WT = 6; WD KO = 6). Mean ± SEM. ROUT 1% method to identify outliers followed by Kruskal-Wallis multiple comparisons test. (E-F) Analysis of the GO “Inflammatory response” from transcriptomes obtained from livers of WT and *Pla2r1* KO mice fed by a CD or a WD. Heatmap (E) and GSEA (F) are shown. (CD WT = 4; CD KO = 4; WD WT = 4; WD KO = 4).

Supplementary Figure 4

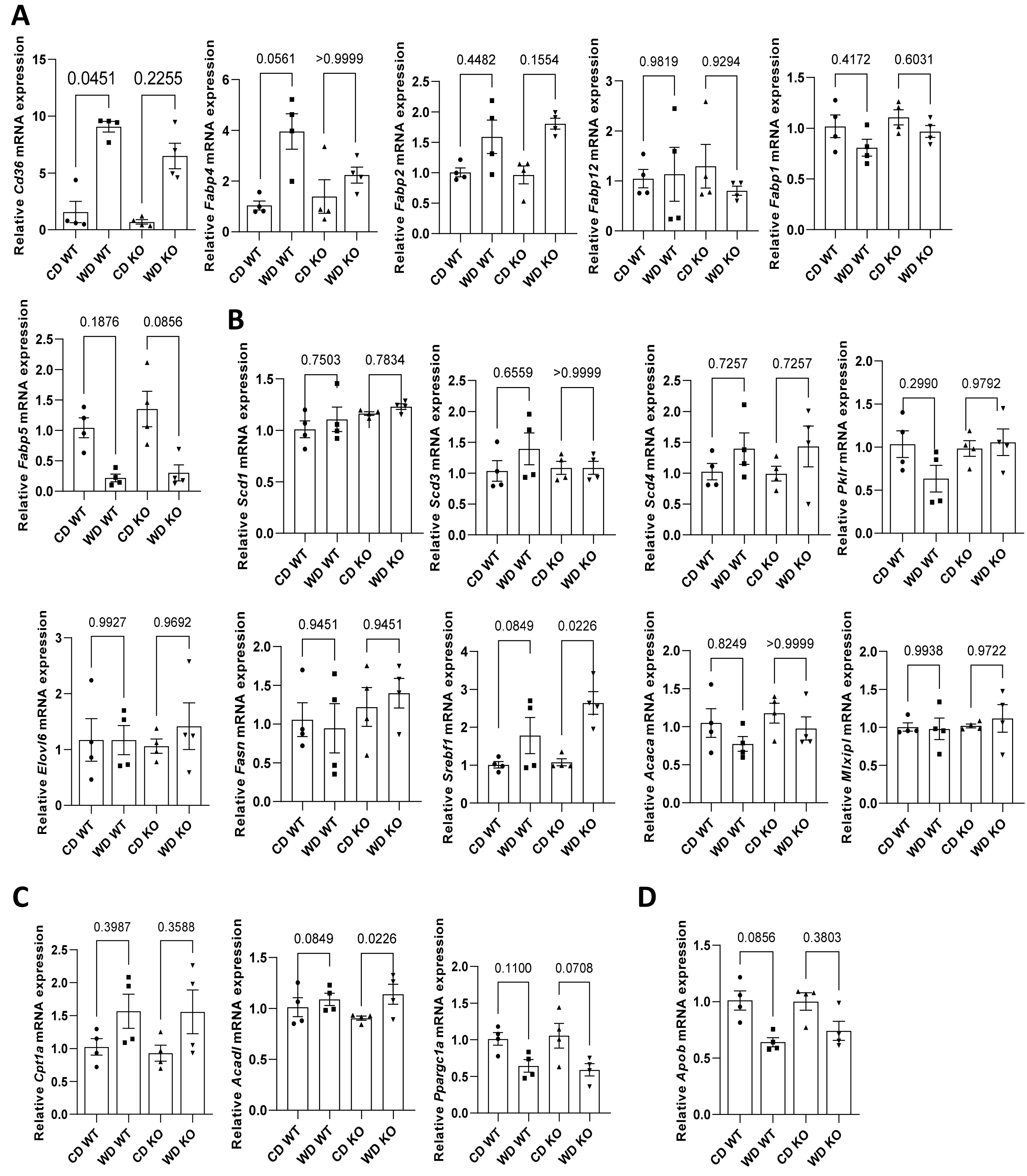

Figure S4 Relative expression, according to transcriptomic analyses, of genes involved in lipid metabolism from liver of wild type (WT) WT or *Pla2r1* KO mice given a chow diet (CD) or a western diet (WD). (A) Absorption of circulating lipids, (B) *de novo* lipogenesis, (C) β-oxidation and (D) lipid export. (CD WT = 4; CD KO = 4; WD WT = 4; WD KO = 4). Mean +/- SEM. Kruskal-Wallis multiple comparisons test.

Supplementary Figure 5

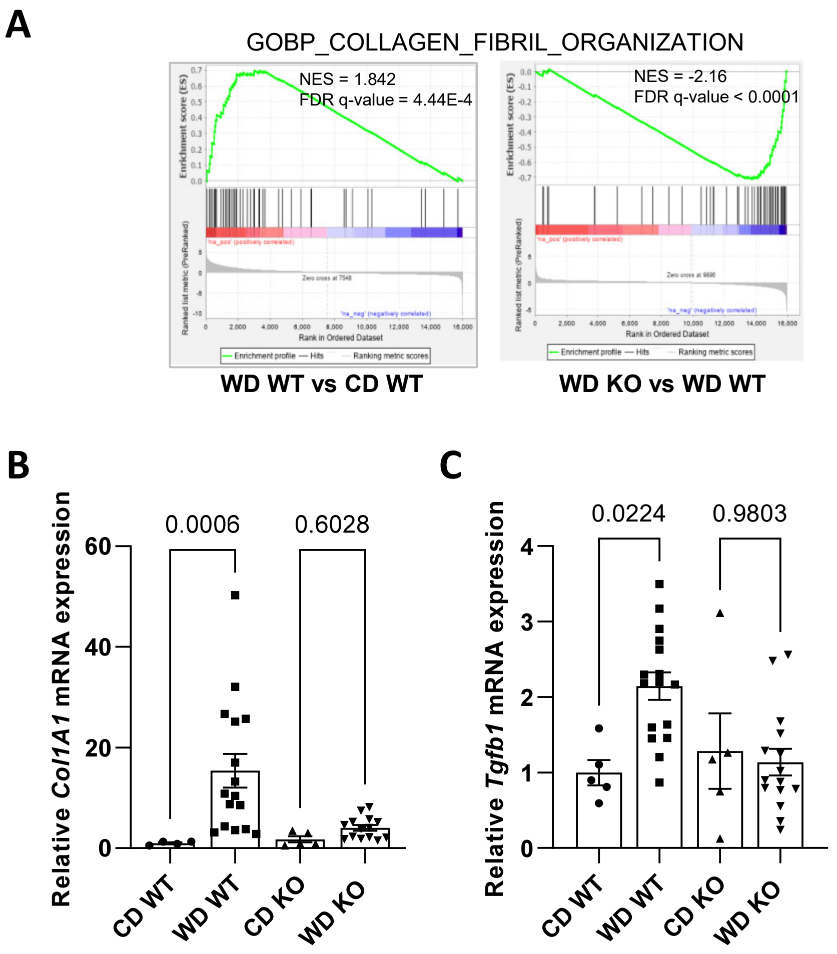

Figure S5 (A) Gene set enrichment analysis showing enrichment of gene sets related to collagen fibril organization from the liver of wild type (WT) or *Pla2r1* KO mice fed a normal Chow diet (CD) or a Western diet (WD) (CD WT = 4; CD KO = 4; WD WT = 4; WD KO = 4) according to transcriptomic analysis. (B) Relative mRNA expression of *Col1a1* of livers from WT or *Pla2r1* KO mice fed a CD or WD (CD WT = 3; CD KO = 5; WD WT = 16; WD KO = 14). Mean ± SEM. Identify outliers followed by Kruskal-Wallis multiple comparisons test. (C) Relative mRNA expression of *Tgfb1* of WT or *Pla2r1* KO mice fed a CD or WD (CD WT = 5; CD KO = 5; WD WT = 16; WD KO = 15). Mean ± SEM. One-way ANOVA multiple comparisons test.

Supplementary Figure 6

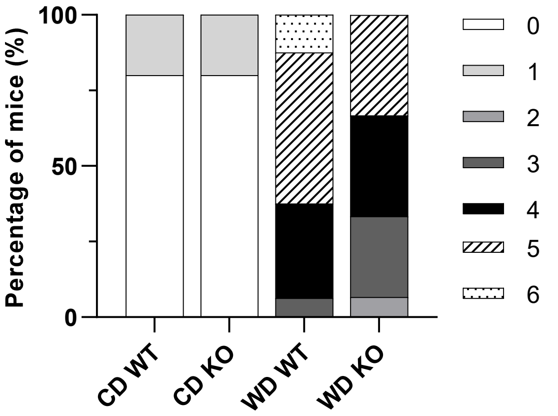

Figure S6 Histograms showing a NASH-like score. Histopathological score from steatosis, fibrosis and ballooning according to Kleiner et al were compiled to generate a NASH-like score. The score was assessed on liver slices stained with H&E or Sirius Red from CD WT (n=5), CD KO (n=5), WD WT (n=16) and WD KO (n=15).

Supplementary Table 1

	3-4-month-old			20-month-old		
	WT	KO	t-test	WT	KO	t-test
ALP UI/L	57.00 ± 16.61	92.7 ± 10.34	0.091	81.25 ± 21.83	40.00 ± 7.17	**0.053**
ALAT UI/L	11.75 ± 5.11	10.50 ± 1.65	0.868	13.20 ± 0.97	16.25 ± 1.78	0.289
ASAT UI/L	95.50 ± 17.5	125.5 ± 15.78	0.4	135.40 ± 22.60	162.0 ± 14.85	0.246
Triglycerides mmol/L	1.30 ± 0.15	1.37 ± 0.08	0.684	0.48 ± 0.08	0.63 ± 0.06	0.184
Cholesterol mmol/L	2.07 ± 0.25	2.42 ± 0.14	0.228	1.93 ± 0.19	1.99 ± 0.14	0.804
Cholesterol HDL mmol/L	1.11 ± 0.22	1.39 ± 0.13	0.278	0.75 ± 0.03	0.71 ± 0.06	0.717
Cholesterol LDL mmol/L	0.19 ± 0.02	0.19 ± 0.02	0.845	0.96 ± 0.15	0.99 ± 0.09	0.842

Table S1 Blood metabolite concentration of 3-4-month-old WT (n=4) and *Pla2r1* KO (n=10) mice, and 20-month-old WT (n=7) and *Pla2r1* KO (n=13) mice. Mean ± SEM. Unpaired t-test.

Supplementary Table 2

	WT		KO		t-test	
	Control diet	Western diet	Control diet	Western diet	CD WT vs WD WT	WD WT vs WD KO
ALP UI/L	69.50 ± 15.90	112.8 ± 11.90	57.80 ± 9.31	66.79 ± 7.89	0.105	**0.004**
ALAT UI/L	6.00 ± 0.00	50.96 ± 13.61	6.00 ± 1.80	29.95 ± 8.25	**0.005**	**0.075**
ASAT UI/L	70.00 ± 22.00	124.00 ± 29.46	59.50 ± 0.50	104.50 ± 12.58	0.283	0.528
Triglycerides mmol/L	0.59 ± 0.20	0.38 ± 0.03	0.80 ± 0.29	0.40 ± 0.03	0.366	0.669
Cholesterol mmol/L	2.31 ± 0.46	3.976 ± 0.49	2.60 ± 0.30	3.76 ± 0.46	0.086	0.752
Cholesterol HDL mmol/L	1.26 ± 0.26	1.87 ± 0.21	1.16 ± 0.28	1.76 ± 0.20	0.155	0.711
Cholesterol LDL mmol/L	0.17 ± 0.01	0.36 ± 0.02	0.22 ± 0.01	0.34 ± 0.03	0.001	0.601

Table S2 Blood metabolite concentration of WT (n=5) and *Pla2r1* KO (n=5) fed with a control Chow Diet and of WT (n=16) and *Pla2r1* KO (n=15) fed with a Western Diet. Mean ± SEM. Unpaired t-test.

Supplementary Table 3

mTel F	CGGTTTGTTTGGGTTTGGGTTTGGGTTTGGGTTTGGGTT
mTel R	GGCTTGCCTTACCCTTACCCTTACCCTTACCCTTACCCT
m36B4 F	ACTGGTCTAGGACCCGAGAAG
m36B4 R	TCAATGGTGCCTCTGGAG ATT
mActin F	CTAAGGCCAACCGTGAAAAG
mActin R	ACCAGAGGCATACAGGGACA
mGapdh F	CCTGCTTCACCACCTTCTTG
mGapdh R	TGTCCGTCGTGGATCTGAC
mTbp F	GGGGAGCTGTGATGTGAAGT
mTbp R	CCAGGAAATAATTCTGGCTCA
mRplp F	GCAGCAGATCCGCATGTCGCTCCG
mRplp R	GAGCTGGCACAGTGACCTCACACGG
mCol1a1 F	CATGTTCAGCTTTGTGGACCT
mCol1a1 R	GCAGCTGACTTCAGGGATGT
mp21 F	TGCGCTTGGAGTGATAGAAA
mp21 R	AACATCTCAGGGCCGAAA
mLpl F	TGCCCTACAAAGTGTTCCATTA
mLpl R	ACTGTGCCGTACAGAGAAATC
mTgfb1 F	TGGAGCAACATGTGGAACTC
mTgfb1 R	GTCAGCAGCCGGTTACCA
mMmp13 F	GTTTGGTCCGATGTAACTCCTC
mMmp13 R	GAAGTCGCCATGCTCCTTAAT
mPlaur F	AGAGACTTTCCTCATTGACTGC
mPlaur R	GTTGCACAGCCTCTTACCATA

Table S3: List of primers
